# Risk Factors, Prognostic Factors, and Nomograms for Distant Metastasis in Patients With Newly Diagnosed Osteosarcoma: A Population-Based Study

**DOI:** 10.3389/fendo.2021.672024

**Published:** 2021-07-30

**Authors:** Bo Chen, Yuan Zeng, Bo Liu, Gaoxiang Lu, Zhouxia Xiang, Jiyang Chen, Yan Yu, Ziyi Zuo, Yangjun Lin, Jinfeng Ma

**Affiliations:** ^1^Department of Spinal Surgery, The Affiliated Hospital of Qingdao University, Qingdao, China; ^2^The First Clinical College, Wenzhou Medical University, Wenzhou, China; ^3^Department of Surgery, The People’s Hospital of Yunhe, Lishui, China

**Keywords:** osteosarcoma, distant metastasis, nomogram, predictor, prognosis

## Abstract

**Background:**

Osteosarcoma is the most common bone cancer, mainly occurring in children and adolescents, among which distant metastasis (DM) still leads to a poor prognosis. Although nomogram has recently been used in tumor areas, there are no studies focused on diagnostic and prognostic evaluation of DM in primary osteosarcoma patients.

**Methods:**

The data of osteosarcoma patients diagnosed between 2004 and 2015 were extracted from the Surveillance, Epidemiology, and End Results (SEER) database. Univariate and multivariate logistic regression analyses were used to identify independent risk factors for DM in osteosarcoma patients, and univariate and multivariate Cox proportional hazards regression analyses were used to determine independent prognostic factors of osteosarcoma patients with DM. We then established two novel nomograms and the results were evaluated by receiver operating characteristic (ROC) curves, calibration curves, and decision curve analysis (DCA).

**Result:**

A total of 1,657 patients with osteosarcoma were included, and 267 patients (16.11%) had DM at the time of diagnosis. The independent risk factors for DM in patients with osteosarcoma include age, grade, T stage, and N stage. The independent prognostic factors for osteosarcoma patients with DM are age, chemotherapy and surgery. The results of ROC curves, calibration, DCA, and Kaplan–Meier (K-M) survival curves in the training, validation, and expanded testing sets, confirmed that two nomograms can precisely predict occurrence and prognosis of DM in osteosarcoma patients.

**Conclusion:**

Two nomograms are expected to be effective tools for predicting the risk of DM for osteosarcoma patients and personalized prognosis prediction for patients with DM, which may benefit clinical decision-making.

## Introduction

Osteosarcoma is the most prevalent form of bone cancer and mainly occurs in children and adolescents (1), which is predominantly derived from the terminus of the long bones, including distal femur (43%), proximal tibias (23%), and proximal humor (10%) (2). Recent reports suggested that the incidence and mortality rates of osteosarcoma have been annually growing by 0.3 and 1.4%, respectively (3, 4). Currently, systemic chemotherapy combined with extensive surgical resection is recognized as the most effective treatment method for osteosarcoma (5–7), and the 5-year survival rate of non-metastatic osteosarcoma patients has been improved to 60–70% with multimodal therapy (8). However, osteosarcoma with distant metastasis (DM) still results in poor prognosis, and only 11–30% of patients can survive with a multimodal combination of surgical resection and chemotherapy (9, 10).

Approximately 20–30% of osteosarcoma patients presented clinical DM (most commonly in the lung) at the time of the first diagnosis (10, 11), and about 25–35% of patients with initially non-metastatic osteosarcoma subsequently develop metastatic diseases (12, 13). Of note, osteosarcoma patients with DM promptly develop more lesions and become resistant to chemotherapy (14), with dismal 5-year overall survival (OS) time less than 20% (15). Therefore, it is imperative to construct exact models to assess the risk of DM of osteosarcoma patients and evaluate the prognosis of patients with DM. Previous studies have revealed that age, M stage, grade, primary tumor site, tumor size, surgery, radiotherapy, and extent of disease were the independent prognostic factors for osteosarcoma (16–18). However, to the best of our knowledge, there are a limited number of studies focusing reliable data on the relationship between clinicopathological features and metastatic pattern of osteosarcoma, and no predictive model for predicting the DM in osteosarcoma or the prognosis of osteosarcoma with DM were established.

Nomogram has been diffusely generated to evaluate the prognosis of cancer patients recently owing to its convenience and precision, which is a good choice for our purpose (19, 20). Thus, we identified a representative cohort from the Surveillance, Epidemiology, and End Results (SEER) database to evaluate incidence, risk factors, and prognosis of *de-novo* metastatic osteosarcoma, and develop two nomograms for predicting the DM in osteosarcoma patients and OS of osteosarcoma patients with DM, respectively.

## Patients and Methods

### Patients

The current study data of osteosarcoma patients were extracted from the SEER database from 2004 to 2015. Inclusion criteria were as follows: (1) Patients were diagnosed with osteosarcoma that was occurring in the bone and joints; (2) Demographic variables, including age, sex, and race were available; (3) Clinical pathological information, including primary tumor site, grade, histological type, TNM, and tumor size were available. Besides, patients diagnosed with autopsy or death certificate were excluded from the study. Finally, 1,657 patients diagnosed with osteosarcoma were included in the present study, including 267 patients who had DM. All patients were used to form a diagnostic cohort to explore the risk factors of DM and develop a predictive nomogram. Moreover, out of 267 osteosarcoma patients with DM, 260 patients with survival time of ≥1 month, and available specific treatment information, including surgery, chemotherapy and radiotherapy, were used to form a prognostic cohort to study the prognostic factors for patients with DM and develop a novel prognostic nomogram.

In the diagnostic cohort, patients were randomly divided into the training (70%), and validation sets (30%) with a ratio of 7:3. As for the prognostic cohort, patients in the training and validation sets were composed of the patients who had DM from corresponding sets in the diagnostic cohort. For each cohort, patients in the training set were used to construct the nomogram, and corresponding patients in the validation set were used to validate it.

### Data Collection

In this study, variables selected to identify the risk factors of DM in osteosarcoma patients are as follows: age, sex, race, primary site, grade, histology type, T stage, N stage, and tumor size. Besides, our research also conducted survival analyses to investigate prognostic factors of osteosarcoma patients with DM. On the basis of the above factors, three treatment variables were included, namely, surgery, radiotherapy, and chemotherapy. In this part, OS was the primary outcome, which was defined as the time interval between the day of diagnosis and the day of death for any reason.

### Statistical Analysis

In the present study, all statistical analysis was performed with SPSS 24.0 and R software (version 3.6.1), and a P value <0.05 (two side) was considered as statistical significance. All osteosarcoma patients were randomly divided into the training and validation sets in R software, and the Chi-square test or Fisher’s exact test was used to compare the distribution of variables between the two sets.

In the diagnostic cohort, the univariate logistic analysis was performed to identify DM-related risk factors. The variables with P <0.05 in the univariate analysis were incorporated into the multivariate logistic analysis with “Forward LR” in SPSS 24.0, to determine independent risk factors of DM in osteosarcoma patients (21). In addition, a novel diagnostic nomogram was built using the “rms” package based on independent risk factors. The receiver operating characteristic (ROC) curve of nomogram and all independent variables were generated, and the corresponding area under the curve (AUC) was calculated to assess the discrimination. Moreover, the calibration curves and decision curve analysis (DCA) were used to evaluate the performance of the nomogram.

For prognostic factors, the univariate Cox regression analysis was applied to determine OS-related factors for osteosarcoma patients with DM. Then, significant variables with P <0.05 were incorporated into the multivariate Cox analysis with “Forward LR” in SPSS 24.0 to further determine the independent prognostic factors. A prognostic nomogram based on the independent prognostic predictors was developed to predict the OS of osteosarcoma patients with DM, and the individual risk score was calculated using the formula of nomogram. In addition, time-dependent ROC curves of nomogram and all independent prognostic variables at 12, 24, and 36 months were generated, and the corresponding time-dependent AUCs were applied to show the discrimination. Calibration curves and DCA of 12, 24, and 36 months were plotted to evaluate the nomogram. According to the median risk score, all osteosarcoma patients with DM were divided into high- and low-risk groups. Kaplan–Meier (K–M) survival curves with the log-rank test were performed to show the difference OS status between the two groups.

## Results

### Baseline Characteristics of the Study Population

A total of 1,657 patients with osteosarcoma were enrolled, and 996 and 661 patients were stratified into the training and validation sets. The mean age of the training and validation sets were 26.69 years old (ranging from 3 to 94) and 26.86 years old (ranging from 3 to 89), respectively. As shown in **Table 1**, the most common primary site location was limb (82.13% in the training set and 79.58% in the validation set), and the most common tumor grades of differentiation were grades III–IV (87.15% in the training set and 86.99% in the validation set). The most common T and N stages were T2 (56.33% in the training set and 53.10% in the validation set) and N0 (97.59% in the training set, and 98.18% in the validation set). Regarding the histological type of osteosarcoma patients, osteosarcoma, NOS accounted for 62.55% in the training set and 63.39% in the validation set. Meanwhile, the Chi-square test proved that the deviation was completely randomized (**Table 1**).

**Table 1:** Baseline clinical characteristics of osteosarcoma patients.

	Training group (n = 996)	Validation group (n = 661)	χ2	P
Age, years			3.049	0.384
≤18	488	326		
19–30	209	122		
31–49	150	117		
≥50	149	96		
Sex			0.315	0.575
Male	533	363		
Female	463	298		
Race			1.250	0.535
Black	165	96		
Other	90	61		
White	741	504		
Primary site			2.372	0.305
Axial	70	47		
Limb	818	526		
Other	108	88		
Grade			0.009	0.925
I–II	128	86		
III–IV	868	575		
Histological type			4.655	0.589
9180	623	419		
9181	154	95		
9182	47	39		
9183	36	26		
9184	4	2		
9185	13	3		
Other	119	77		
T			3.871	0.144
T1	404	296		
T2	561	351		
T3	31	14		
N			0.660	0.417
N0	972	649		
N1	24	12		
M			0.375	0.540
M0	840	550		
M1	156	111		
Tumor size, mm			1.862	0.394
<50	131	98		
50–100	463	316		
>100	402	247		

9180, Osteosarcoma, NOS; 9181, Chondroblastic osteosarcoma; 9182, Fibroblastic osteosarcoma; 9183, Telangiectatic osteosarcoma; 9184, Osteosarcoma in Paget disease of bone; 9185, Small cell osteosarcoma.

### Incidence and Risk Factors of Distant Metastasis in Osteosarcoma Patients

A total of 267 cases (16.11%) confirmed as DM at initial diagnosis and 1,390 cases (83.89%) without it. As shown in **Table 2**, nine potential factors were analyzed by the univariate logistic analysis, and the result revealed six DM-related variables, including age, primary site, grade, T stage, N stage, and tumor size. Additionally, the multivariate logistic regression analysis determined that patients younger than 18 or older than 50, higher T stage, higher N stage, and higher grade were independent risk predictors of DM in primary osteosarcoma patients (**Table 2**).

**Table 2:** Univariate and multivariate logistic analyses of distant metastasis in osteosarcoma patients.

	Univariate analysis			Multivariate analysis		
	OR	95%CI	P	OR	95%CI	P
Age, years						
≤18	Reference			Reference		
19–30	0.456	0.279–0.748	0.002	0.499	0.299–0.832	0.008
31–49	0.190	0.086–0.418	0.000	0.246	0.110–0.548	0.001
≥50	0.859	0.536–1.376	0.526	1.042	0.632–1.720	0.871
Race						
Black	Reference					
Other	0.905	0.417–1.963	0.801			
White	1.294	0.794–2.109	0.302			
Sex						
Female	Reference					
Male	1.261	0.892–1.782	0.189			
Primary site						
Axial	Reference					
Limb	0.882	0.470–1.656	0.696			
Other	0.258	0.093–0.715	0.009			
Grade						
I–II	Reference			Reference		
III–IV	6.581	2.395–18.086	<0.001	3.723	1.324–10.470	0.013
Histological type						
9180	Reference					
9181	0.760	0.463–1.248	0.279			
9182	0.203	0.048–0.848	0.029			
9183	1.101	0.471–2.577	0.824			
9184	4.562	0.636–32.736	0.131			
9185	1.369	0.371–5.054	0.638			
Other	0.329	0.156–0.693	0.003			
T						
T1	Reference			Reference		
T2	2.155	1.455–3.191	<0.001	2.015	1.337–3.036	0.001
T3	5.911	2.670–13.084	<0.001	4.318	1.856–10.044	0.001
N						
N0	Reference			Reference		
N1	6.851	3.010–15.592	<0.001	6.018	2.503–14.468	<0.001
Tumor size, mm						
<50	Reference					
50–100	2.466	1.151–5.285	0.020			
>100	4.061	1.910–8.636	<0.001			

9180, Osteosarcoma, NOS; 9181, Chondroblastic osteosarcoma; 9182, Fibroblastic osteosarcoma; 9183, Telangiectatic osteosarcoma; 9184, Osteosarcoma in Paget disease of bone; 9185, Small cell osteosarcoma; OR, Odds ratio; CI, Confidence interval.

### Diagnostic Nomogram Development and Validation

**Figure 1:**
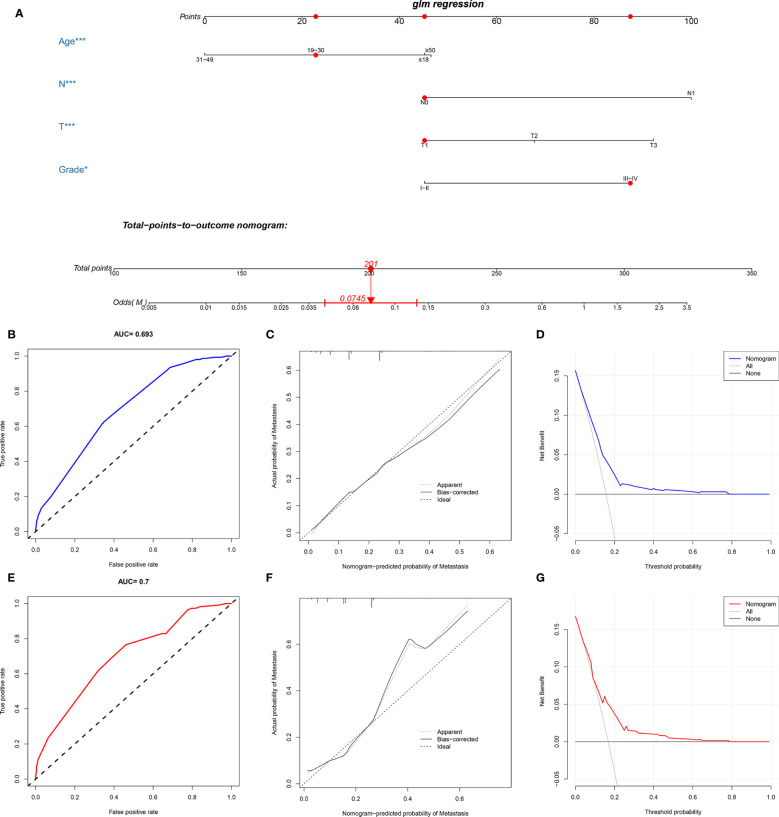
Construction and validation of a diagnostic nomogram. A nomogram to estimate the risk of DM in osteosarcoma patients **(A)**. The receiver operating characteristic curve **(B)**, calibration curve **(C)**, and decision curve analysis **(D)** of the training set, and the receiver operating characteristic curve **(E)**, calibration curve **(F)**, and decision curve analysis **(G)** of the validation set.

**Figure 2:**
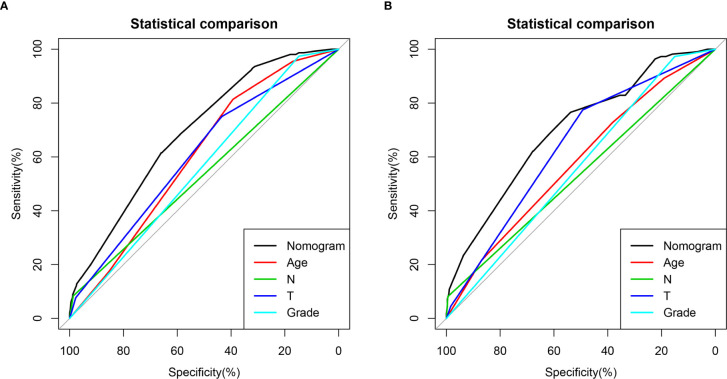
Comparison of area under the receiver operating characteristic curves between nomogram and all independent factors, including Grade, T stage, N stage, and Age in the training set **(A)** and Validation set **(B)**.

A novel nomogram for predicting the risk of DM in osteosarcoma patients was established based on the four independent predictors (**Figure 1A**). Then, we established the ROC curves of the training and validation sets, and the AUCs of the nomogram were 0.693 and 0.700 in the training and validation set, respectively (**Figures 1B, E**). Meanwhile, the ROC curves of all independent predictors were also generated (**Figures 2A, B**), demonstrating a better discriminative ability than the other individual factors, both in the training and validation sets. More importantly, the calibration curves of the nomogram illustrated excellent consistency between the observed and predicted results (**Figures 1C, F**). As shown in **Figures 1D, G**, DCA curves indicated that the diagnostic nomogram can serve as a precise tool for DM assessment. In addition, to further verify the applicability of the model in the absence of external data, we went back to the SEER database and re-screened suitable patients with complete age, T stage, N stage, grade stage, and M stage. Totally, 1,667 patients were obtained to form an expanded testing set. Meanwhile, the ROC curve showed that the AUC of the nomogram was 0.696 in the expanded testing set (**Supplementary Figure 1A**), and calibration, DCA, and ROC curves of all independent factors (**Supplementary Figures 1B–D**) also proved the good performance of the diagnostic nomogram.

### Prognostic Factors for Osteosarcoma Patients With DM

In the present study, 260 eligible osteosarcoma patients with DM were used to explore prognostic factors. As shown in **Table 3**, 207 (79.61%) patients underwent surgery, 48 (18.46%) underwent radiotherapy, and 240 (92.31%) underwent chemotherapy. The Chi-square test and Fisher’s exact test indicated that the differences of all variables were not significant between the training set and the validation set. Then, the univariate and multivariate Cox regression analyses were used to screen robust prognostic factors, which revealed that the higher age (P <0.001), absence of surgery (P <0.001) and absence of chemotherapy (P = 0.001) were independent prognostic factors for osteosarcoma patients with DM (**Table 4**).

**Table 3:** Baseline clinical characteristics of patients diagnosed as osteosarcoma with DM.

	Training group (n = 154)	Validation group (n = 106)	χ2	P
Age, years			7.178	0.066
≤18	100	54		
19–30	22	18		
31–49	6	11		
≥50	26	23		
Sex			1.664	0.197
Male	91	71		
Female	63	35		
Race			1.968	0.374
Black	22	21		
Other	10	9		
White	122	76		
Primary site			3.557	0.169
Axial	13	17		
Limb	135	85		
Other	6	4		
Grade			0.245	0.621
I–II	4	1		
III–IV	150	105		
Histological type			8.534	0.171
9180	110	83		
9181	22	14		
9182	2	5		
9183	7	1		
9184	2	0		
9185	3	1		
Other	8	2		
T			1.500	0.472
T1	39	23		
T2	103	79		
T3	12	4		
N			0.005	0.942
N0	142	98		
N1	12	8		
Tumor size			1.279	0.528
<50	8	6		
50–100	63	35		
>100	83	65		
Surgery	128	78	3.467	0.063
Radiotherapy	27	21	0.217	0.642
Chemotherapy	142	98	0.005	0.942

9180, Osteosarcoma, NOS; 9181, Chondroblastic osteosarcoma; 9182, Fibroblastic osteosarcoma; 9183, Telangiectatic osteosarcoma; 9184, Osteosarcoma in Paget disease of bone; 9185, Small cell osteosarcoma.

**Table 4:** Univariate and multivariate Cox analyses in osteosarcoma patients with DM.

	Univariate analysis			Multivariate analysis		
	HR	95%CI	P	HR	95%CI	P
Age, years						
≤18	Reference			Reference		
19–30	1.993	1.148-3–460	0.014	1.779	1.018–3.108	0.043
31–49	2.223	0.798–6.193	0.127	2.272	0.813–6.355	0.118
≥50	6.470	3.858–10.851	<0.001	4.477	2.480–8.082	<0.001
Race						
Black	Reference					
Other	0.947	0.341–2.631	0.917			
White	1.135	0.641–2.008	0.664			
Sex						
Female	Reference					
Male	1.064	0.704–1.608	0.767			
Primary site						
Axial	Reference					
Limb	0.334	0.181–0.619	<0.001			
Other	0.571	0.161–2.027	0.386			
Grade						
I–II	Reference					
III–IV	0.739	0.234–2.334	0.606			
Histological type						
9180	Reference					
9181	1.201	0.684–2.107	0.523			
9182	0.761	0.105–5.496	0.787			
9183	1.640	0.708–3.799	0.248			
9184	4.571	1.102–18.960	0.036			
9185	3.526	1.088–11.430	0.036			
Other	0.328	0.080–1.340	0.121			
T						
T1	Reference					
T2	0.865	0.540–1.386	0.546			
T3	1.146	0.532–2.469	0.727			
N						
N0	Reference					
N1	1.729	0.868–3.443	0.119			
Tumor size						
<50	Reference					
50–100	0.564	0.237–1.346	0.197			
>100	0.705	0.303–1.638	0.416			
Surgery						
No	Reference			Reference		
Yes	0.185	0.113–0.303	<0.001	0.226	0.134–0.382	<0.001
Chemotherapy						
No	Reference			Reference		
Yes	0.255	0.135–0.481	<0.001	0.485	0.238–0.990	0.047
Radiotherapy						
No	Reference					
Yes	2.340	1.430–3.829	0.001			

9180, Osteosarcoma, NOS; 9181, Chondroblastic osteosarcoma; 9182, Fibroblastic osteosarcoma; 9183, Telangiectatic osteosarcoma; 9184, Osteosarcoma in Paget disease of bone; 9185, Small cell osteosarcoma; HR, Hazard ratio; CI, Confidence interval.

### Prognostic Nomogram Development and Validation

**Figure 3:**
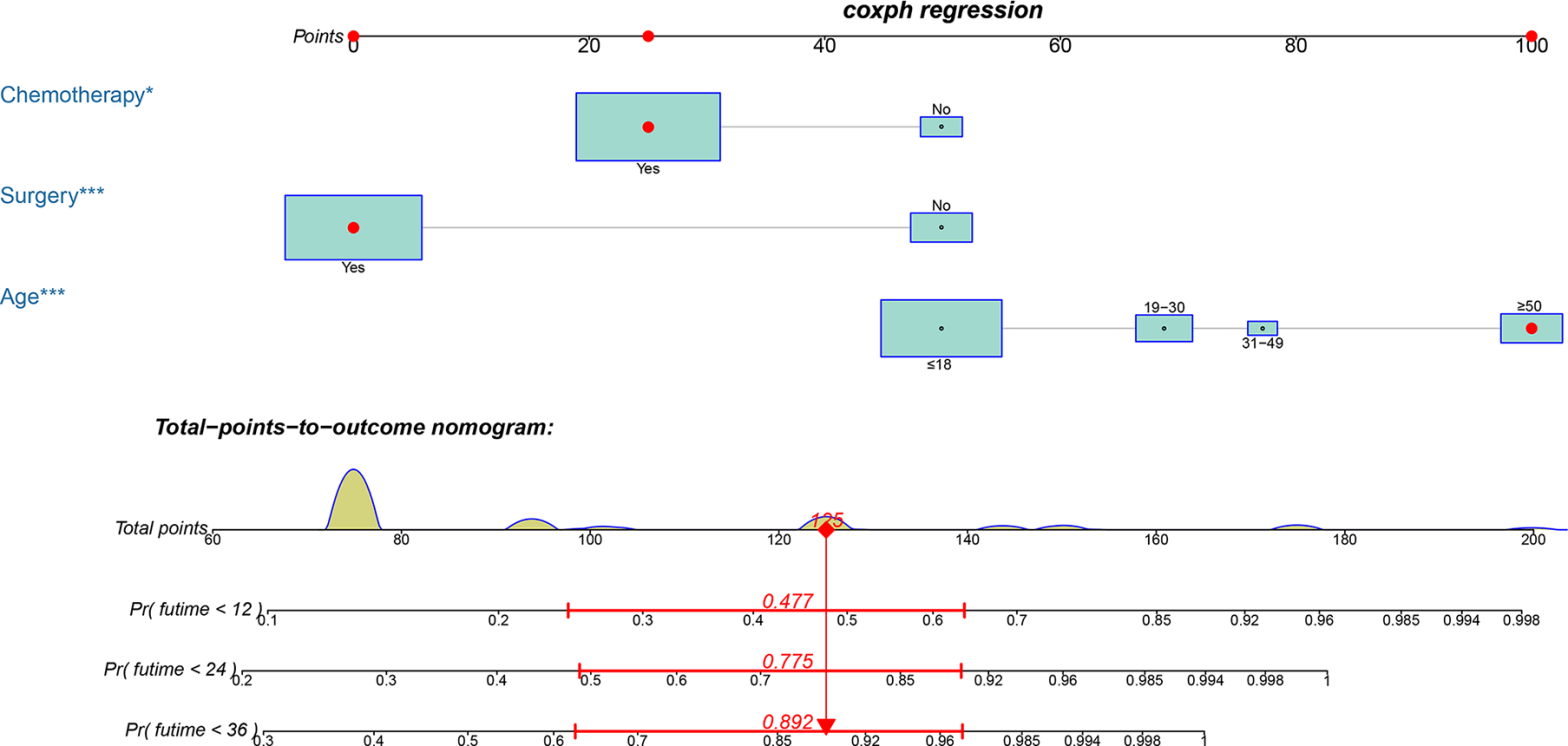
A prognostic nomogram for predicting the OS of osteosarcoma patients with DM for the 12, 24, and 36 months.

**Figure 4:**
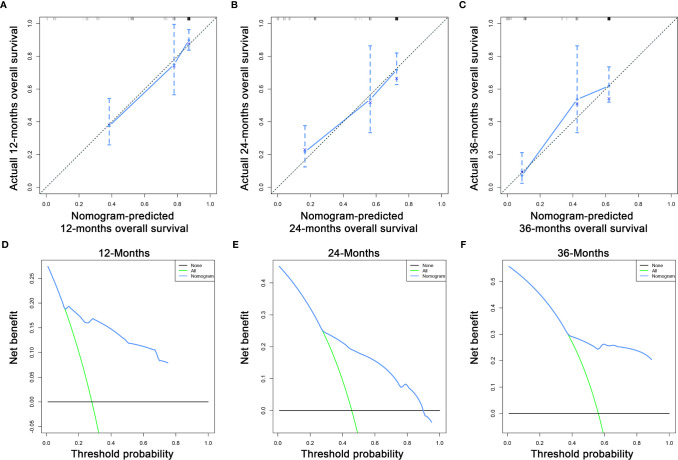
The calibration curves of the nomogram for the 12 **(A)**, 24 **(B)**, and 36 months **(C)** in the training set. The decision curve analysis of the nomogram for the 12 **(D)**, 24 **(E)**, and 36 months **(F)** in the training set.

**Figure 5:**
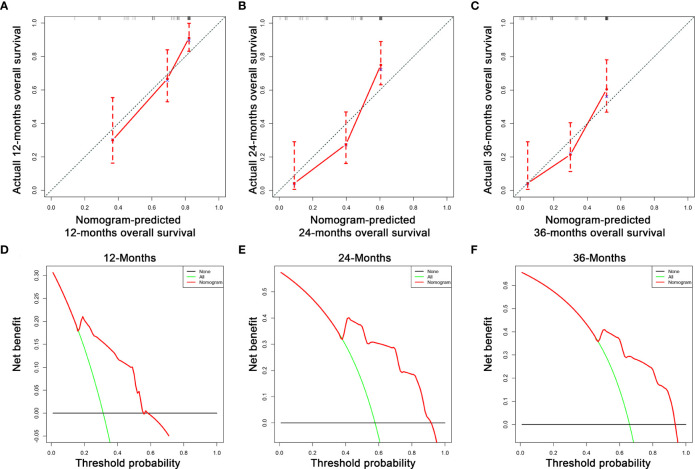
The calibration curves of the nomogram for the 12 **(A)**, 24 **(B)**, and 36 months **(C)** in the validation set. The decision curve analysis of the nomogram for the 12 **(D)**, 24 **(E)**, and 36 months **(F)** in the validation set.

**Figure 6:**
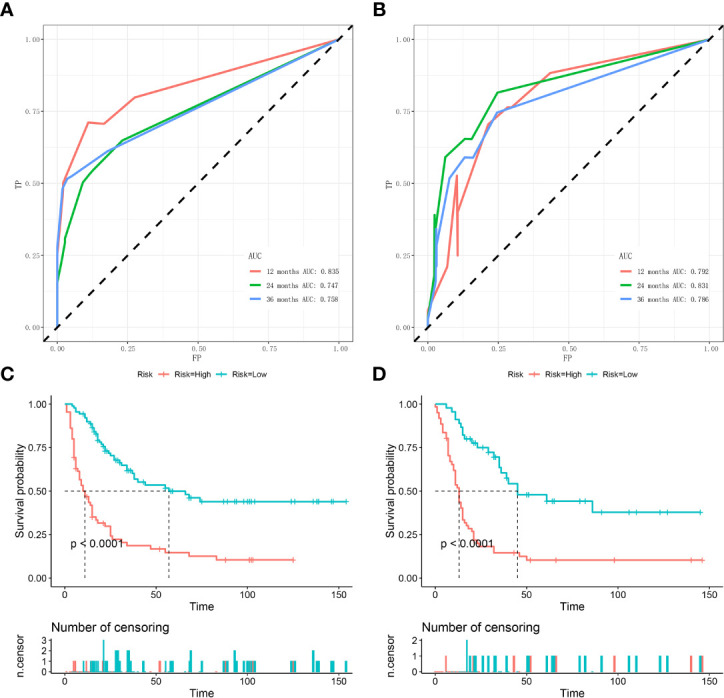
Time-dependent ROC curve analysis of the nomogram for the 12, 24, and 36 months in the training set **(A)** and the validation set **(B)**. The Kaplan–Meier survival curves of the patients in the training set **(C)** and in the validation set **(D)**.

**Figure 7:**
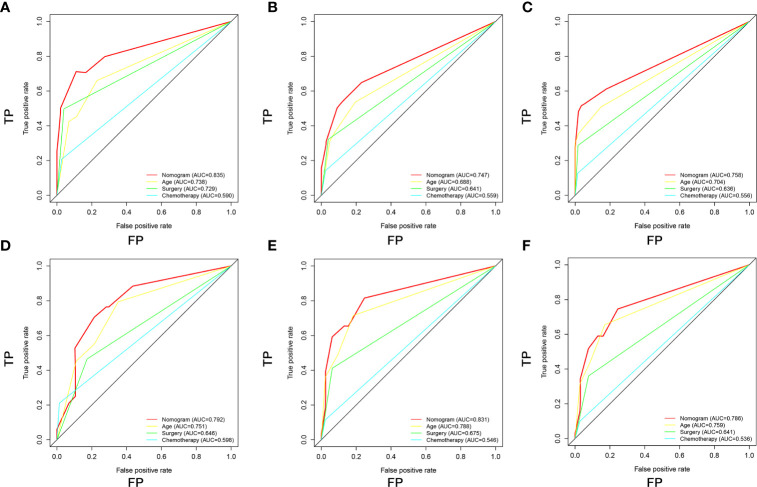
Comparison of area under the receiver operating characteristic curves between nomogram and all independent factors, including Age, Surgery, and Chemotherapy for the 12 **(A, D)**, 24 **(B, E)**, and 36 **(C, F)** months in the training set and validation set.

Based on the three prognostic factors, a nomogram was established to predict the OS of osteosarcoma patients with DM (**Figure 3**). The calibration curves of the nomogram for the probability of 12, 24, and 36 months OS exhibited a strong agreement between nomogram-predicted OS and the actual outcome in the training set (**Figures 4A–C**) and validation set (**Figures 5A–C**). Additionally, the DCA curves also determined that the nomogram had good performance in clinical practice (**Figures 4D–F**, **5D–F**). Moreover, ROC analysis showed that the AUCs of the nomogram in the training set for the 12, 24, and 36 months reached 0.835, 0.747, and 0.758 (**Figure 6A**), and 0.792, 0.831, and 0.786, respectively, in the validation set (**Figure 6B**), which also showed good discrimination in predicting the OS of osteosarcoma patients with DM. The K–M curves indicated that the patients in the high-risk group had significantly worse OS than the patients in the low-risk group (**Figures 6C, D**). Furthermore, we further compared the discrimination between nomogram and each independent prognostic factor, and the results indicated that the discrimination of nomogram was better than all independent prognostic factors at 12, 24, and 36 months (**Figures 7A–F**). Meanwhile, although histology type was not an independent prognostic factor for osteosarcoma patients with DM, considering histologically different osteosarcomas arising from different cells may affect the predictive ability of the nomogram, the stratification analysis was implemented to evaluated this. Due to the limitation of the study sample, we only divided the patients into two subgroups (9180: osteosarcoma, Nos Vs others). As shown in **Supplementary Figure 2**, the AUCs of patients with 9,180 for predicting the 12, 24, and 36 months OS were 0.849, 0.755, and 0.756 in the training set and 0.786, 0.855, and 0.810 in the validation set (**Supplementary Figures 2A, C**), and ROC analysis showed the AUCs of patients with other histology type reached 0.821, 0.732, and 0.761 in the training set and 0.817, 0.706, and 0.640 in the validation set (**Supplementary Figures 2B, D**), which implied the prognostic nomogram could serve a rigorous tool for patients with different histology type.

### Validating the Prognostic Nomogram in an Expanded Testing Set

**Figure 8:**
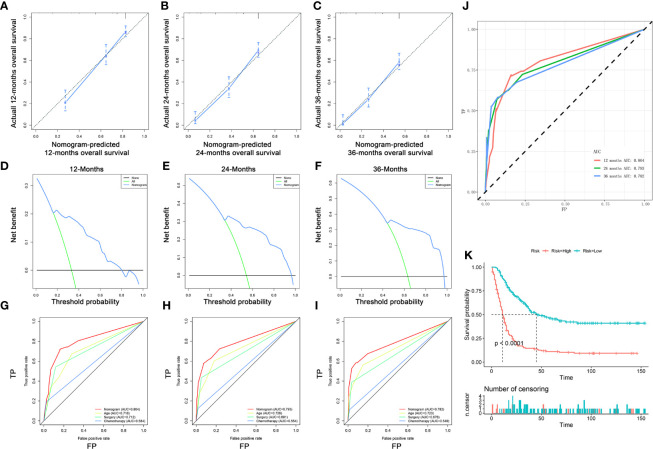
Validating the prognostic nomogram in the expanded testing set. The calibration curves of the nomogram for the 12 **(A)**, 24 **(B)**, and 36 months **(C)** in the expanded testing set. The decision curve analysis of the nomogram for the 12 **(D)**, 24 **(E)**, and 36 months **(F)** in the expanded testing set. Comparison of area under the receiver operating characteristic curves between nomogram and all independent factors for the 12 **(G)**, 24 **(H)**, and 36 **(I)** months in the expanded testing set. **(J)** Time-dependent ROC curve analysis of the nomogram for the 12, 24, and 36 months in the expanded testing set. **(K)** The Kaplan–Meier survival curve of the patients in the expanded testing set.

A total of 363 patients with DM with complete age, chemotherapy, and surgery information from the SEER database were enrolled to form an expanded testing set. In the expanded testing set, the favorable calibration plots of the prognostic nomogram implied that OS of patients with DM predicted by the nomogram were highly consistent with the actual observation (**Figures 8A–C**). Additionally, DCA was performed and the results proved that the prognostic nomogram can serve as an effective clinical tool (**Figures 8D–F**). Also, the discrimination of nomogram was better than three independent predictors in 12, 24, and 36 months (**Figures 8G–I**). Moreover, the AUCs of patients for 12, 24, and 36 months OS prediction were 0.804, 0.793, and 0.782 (**Figure 8J**), and the results of the K–M survival analysis suggested that there existed different survival patterns among patients in the high- and low-risk groups (**Figure 8K**).

## Discussion

Osteosarcoma is an aggressive tumor of bone and prone to DM, occurring in 15–40% of patients (22, 23). Almost all deaths in patients with osteosarcoma are caused by DM (24, 25). Once osteosarcoma patients develop DM, the OS decreases dramatically and the 5-year survival rate decreases to 20% (15, 26). The reason of poor prognosis of advanced osteosarcoma patients is that patients with DM could not benefit much from surgery, chemotherapy, and novel immunotherapy (6, 27). Therefore, we must identify the effective risk and prognostic factors for osteosarcoma patients with DM to diagnose at early stage, facilitate the early prevention, and evaluate the prognosis of osteosarcoma patients with DM. In the present study, we constructed a diagnostic nomogram for predicting the DM in newly diagnosed osteosarcoma patients, and a prognostic nomogram for patients with DM. By obtaining the data of several key accessible variables on the nomograms, diagnosis-related and prognosis-related scores can be calculated, which can provide guidance for further clinical evaluation and intervention.

Recently, there are many studies focused on DM in osteosarcoma, but most of them are performed at the molecular level rather than clinicopathologic features. The expression of chemokine receptor CXCR3 (28), lncRNA HNF1A-AS1 (29), and miR-206 (30) were identified to be associated with DM in osteosarcoma patients, and m6A-related signature (31), and tumor microenvironment (TME)-related signature (32) were constructed to have an early detection of DM. However, we should point out that the sample size of these studies was usually small and they were single-center studies that lacked sufficient validation, which caused these biomarkers unpractical and difficult to apply immediately to clinical management. Moreover, as for clinical characteristics research, Miller et al. determined that advanced age, tumor in the axial skeleton, larger tumor size, and residence in a less affluent county were independent predictors of metastatic disease in osteosarcoma patients (33). Another similar study that focusing on osseous neoplasms (including osteosarcoma) found that higher tumor grade, Ewing sarcoma and osteosarcoma, and larger tumor size were associated with an increased risk of lung metastasis (34). In this study, we incorporated the latest large samples with comprehensive clinical information from the SEER database and find that the incidence of DM was 16.11%, which was lower than the previous study. Four significant predictors for DM in osteosarcoma patients were determined, namely, age, N stage, T stage, and Grade. The association between TNM stage and DM in osteosarcoma patients has been confirmed in the previous studies (35, 36). However, it was unexpected that patients with age younger than 18 or older than 50 are more likely to have metastasis disease. We speculated that it may be caused by physical development status, while children’s bodies are not yet fully developed and old people are decaying. The children’s immune systems are not fully mature, and aging is accompanied by cell aging, including changes of protein, metabolism, and nuclear genome instability (37, 38), which may be involved in the occurrence and progression of tumors.

As the prognosis is extremely poor in osteosarcoma patients with DM, the early discovery of DM is crucial for patients to receive appropriate surgical resection and chemotherapy (39). To date, most studies stopped at independent risk factors and only one realistic model has been established to predict the risk of DM in osteosarcoma patients. In the similar predictive tool established by Li et al. (40), surgery, a post-diagnosis treatment was included in the nomogram for the diagnosis of DM. This sequential relationship was reverse and irrational, resulting in the model’s uselessness. To address this inadequacy, we developed a novel diagnostic nomogram based on four independent predictors, and the excellent performance was demonstrated by calibration curves, ROC curves, and DCA, which may improve the current situation of risk assessment and make the individualized clinical decision more accurate.

Although osteosarcoma patients with DM dramatically develop more lesions and become resistant to chemotherapy (14), underscoring a critical need for new treatments strategies, continuous chemotherapy still plays an important role in prolonging patients’ life and several clinical trials are still ongoing (41, 42). Surgery alone, the only effective way to treat osteosarcoma decades ago, which consisted of removing the tumor of amputating, didn’t reduce mortality below 80%, but there is still a place for osteosarcoma patients with DM (1). Interestingly, our results showed that the absence of surgery and chemotherapy had a significant negative impact on the OS, which is consistent with the above results. Radiotherapy had no significant effect on prognosis, which was consistent with the previous study (43). Moreover, it is generally believed that osteosarcoma patients with DM with higher age had a poorer OS prognosis than younger patients (44). Our study showed that patients with higher age are more likely to have poorer OS. Notably, we constructed a novel prognostic nomogram to predict the prognosis of osteosarcoma patients with DM, and the discrimination of nomogram was confirmed higher than any independent predictors, which indicated that the nomogram may open up a new prospect for personalized assessment and clinical decision-making. Although some predictive nomograms have been established in previous studies, we think our study improves upon the previous work. In the comprehensive nomogram based on gene signature and clinical predictors established by Fu et al. and Zhang et al. (45, 46), using gene signature to predict prognosis is more costly and less convenient than our nomogram with only three simple clinical variables. Additionally, the advantages of our study compared with the existing prognostic nomograms (18, 47–50) are as follow. First, we do not study the same subjects as they do. Jiang et al. used juvenile osteosarcoma as a research object (47), Gao et al. used chondroblastic osteosarcoma as a study object (48), He et al. studied patients with osteosarcoma as secondary malignancy (49), and Zhang et al. and Yang et al. made all osteosarcoma patients as study objects (18, 50), while we selected the patient with DM who lacked effective treatment and had a poor prognosis as a research object, which is more specific clinically and has not been studied. Second, our research included fewer clinical variables and had comparable or better AUC values. Third, all in the absence of external data, our study implemented more adequate verification tools, and went back to the SEER database to verify the performance of the nomogram again.

Nevertheless, we should acknowledge that this study has some shortcomings. First, the limited number of osteosarcoma patients with DM (N = 267) may have contributed to the possible error. Second, although our nomograms were constructed in the training set and validated in the validation and expanded testing sets, no available publicly osteosarcoma data in other database was enrolled, which has an inherent bias. Third, the information collected in the SEER database was about the disease at the time of initial diagnosis, which meant that the DM which occurred in the latter stage cannot be included. Additionally, although race has no effect on osteosarcoma DM and prognosis of patients with DM, most of our subjects were white, which makes the applicability of our models to other ethnic groups unknown and requires further study. Finally, we did not have specific information about systemic treatments, and this was a retrospective study and only patients with complete information were recorded.

## Conclusions

Our study determined that age, N stage, T stage, and grade stage were the independent risk factors of DM for osteosarcoma, and age, surgery, and chemotherapy were the independent prognostic factors for the patients with DM. Two nomograms could be used as an intuitive graphic tool in osteosarcoma to quantitatively evaluate the risk and prognosis of osteosarcoma with DM, and guide the clinical decision-making.

## Data Availability Statement

The dataset from SEER database generated and/or analyzed during the current study are available in the SEER dataset repository (https://seer.cancer.gov/).

## Author Contributions

BC, YZ, and JFM conceived of and designed the study. GXL, ZXX, BL and JYC performed literature search. BC, BL and YY generated the figures and tables. BC, ZYZ and YJL analyzed the data. YZ, and BL wrote the manuscript and BC critically reviewed the manuscript. JFM supervised the research. All authors have read and approved the manuscript. BC, YZ and BL contributed equally to this work.

## Conflict of Interest

The authors declare that the research was conducted in the absence of any commercial or financial relationships that could be construed as a potential conflict of interest.

## Publisher’s Note

All claims expressed in this article are solely those of the authors and do not necessarily represent those of their affiliated organizations, or those of the publisher, the editors and the reviewers. Any product that may be evaluated in this article, or claim that may be made by its manufacturer, is not guaranteed or endorsed by the publisher.
