# Supplementary figures and images for: Risk Factors, Prognostic Factors, and Nomograms for Distant Metastasis in Patients With Newly Diagnosed Osteosarcoma: A Population-Based Study
Source: Front Endocrinol (Lausanne). 2021 Jul 30;12:672024. doi: 10.3389/fendo.2021.672024 (PMC8362092; doi:10.3389/fendo.2021.672024)

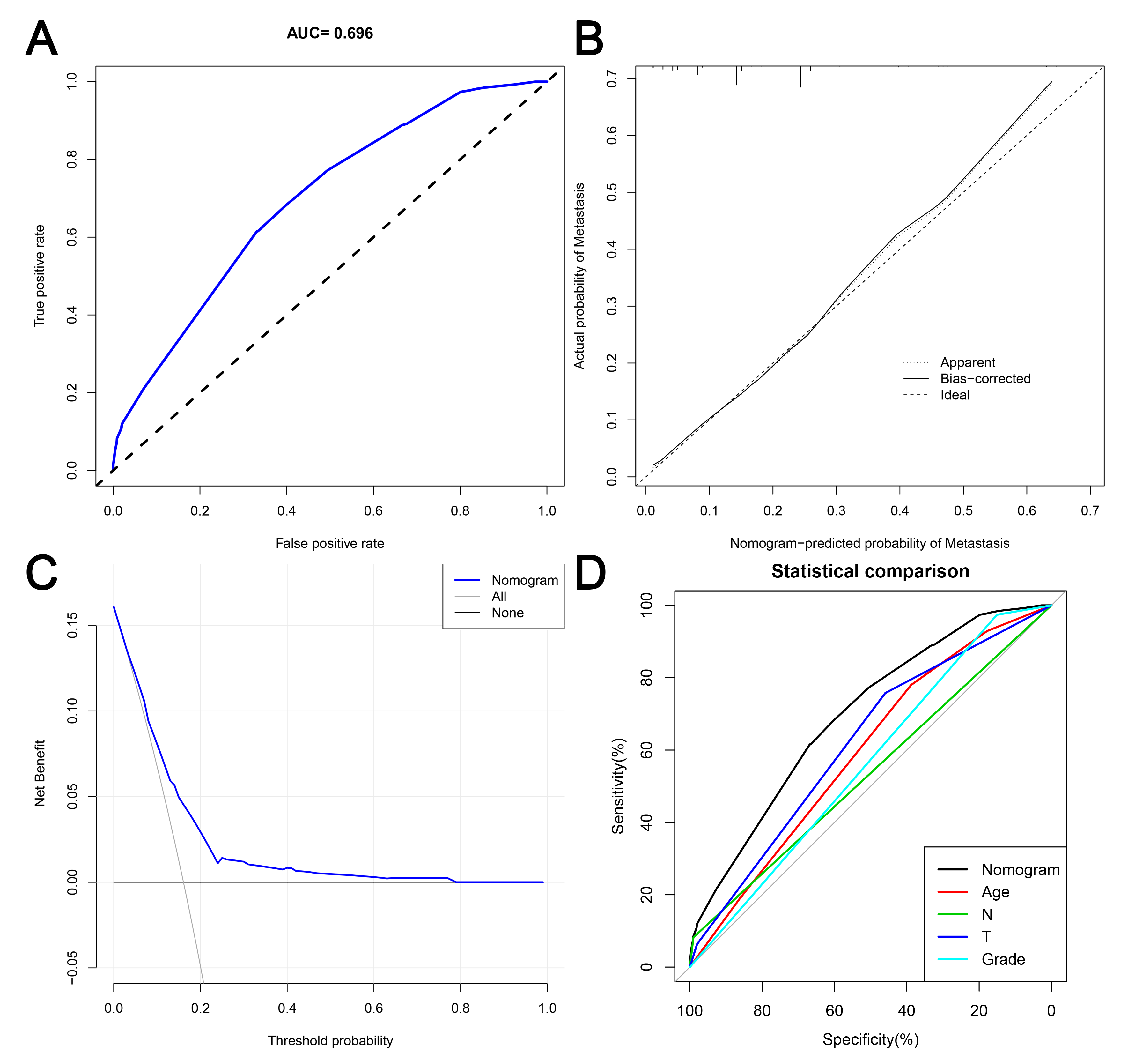

Supplement: Supplementary file 1 [file Image_1.tif]

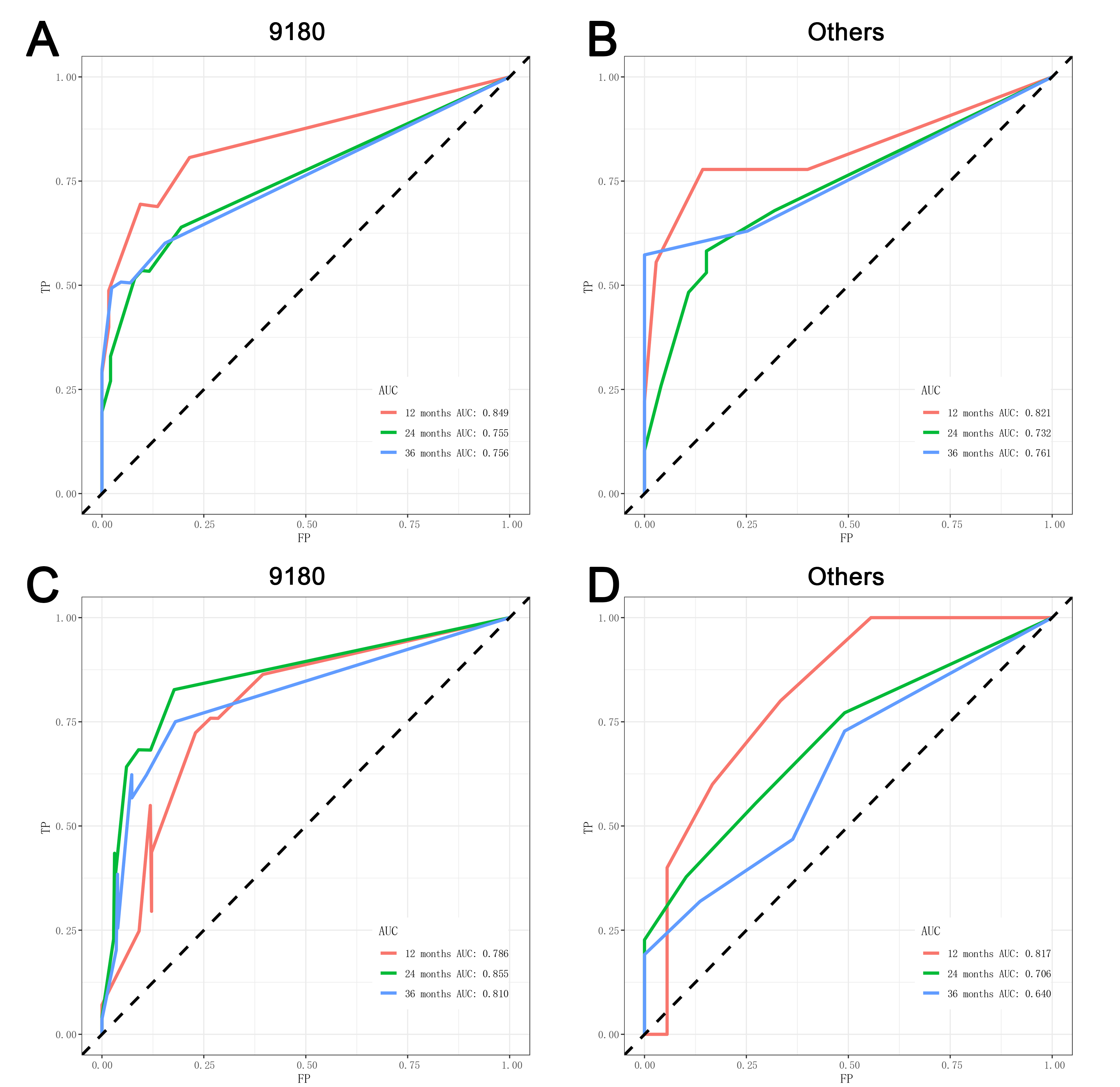

Supplement: Supplementary file 2 [file Image_2.tif]
